# Effect of temperature in the degradation of cannabinoids: From a brief residence in the gas chromatography inlet port to a longer period in thermal treatments

**DOI:** 10.3389/fchem.2022.1038729

**Published:** 2022-11-01

**Authors:** María Teresa García-Valverde, Carolina Sánchez-Carnerero Callado, Maríadel Carmen Díaz-Liñán, Verónica Sánchez de Medina, Jesús Hidalgo-García, Xavier Nadal, Lumír Hanuš, Carlos Ferreiro-Vera

**Affiliations:** ^1^ Phytoplant Research S.L.U, Córdoba, Spain; ^2^ Institute for Drug Research, School of Pharmacy, Faculty of Medicine, Hebrew University, Ein Kerem Campus, Jerusalem, Israel

**Keywords:** cannabis, by-products, thermal degradation, gas chromatography, kinetics

## Abstract

The substantial increase in legalization and subsequent regulation of cannabis has intensified the control and analytical monitoring of cannabis products to assure sample quality and control the cannabinoid content of the crop. In this sense, the restriction on cultivating legal cannabis plants has been limited to 0.2–0.3% of Δ^9^-THC content, depending on the host country’s laws. Thereby, cannabis flowers containing more than this limit are considered illicit drug-type cultivations and require the obtention of specific permits to work with them. The official method established by the European Commission set the gas chromatography/flame ionization detector (GC-FID) as the proper instrument to analyze the delta-9 tetrahydrocannabinol (Δ^9^-THC) content. In the present work, the potential drawbacks associated with the utilization of the official method for the evaluation of the Δ9-THC content have been described. Thus, the effect of the GC injector port temperature in the degradation of cannabinoids was evaluated, observing the degradation of CBD by 20%, generating Δ^9^-THC and CBN as by-products. Likewise, 17.2% of Δ^9^-THC was degraded, producing CBN as a by-product. Therefore, despite the brief residence of cannabinoids in the GC inlet, the effect of temperature is noteworthy and must be considered. Derivatization of cannabinoids should be a mandatory step to prevent the thermal degradation of cannabinoids, assuring the accuracy of the results. Furthermore, the evaluation of cannabinoid degradation thermally treated for longer periods of time was carried out. The kinetic degradation of CBD was evaluated in this way, observing a degradation of 0.22 μg/L per second. At the same time, the kinetics of the appearance of Δ^9^-THC demonstrates the intermediate nature of this cannabinoid, being degraded at 0.03 s^−1^ μM^−1^. The degradation of CBD also produced CBN and CBE as by-products.

## 1 Introduction

Cannabis has been the subject of controversy for many years due to the negative connotation related to its use for recreational purposes, owing to the psychotropic effects produced by some of its constituents, which have adversely affected this industry. Nevertheless, the benefits of this plant are known worldwide, and its potential has been exploited for the treatment of many illnesses. For this reason, the legalization and regulation of cannabis for medical and recreational purposes has experienced a considerable increase in many countries, including Uruguay, Canada, Israel, Australia, Jamaica, and Columbia. In 1976, the content of delta-9 tetrahydrocannabinol (Δ^9^-THC) was limited to 0.3%, although this restriction may vary among countries (Sgrò et al., 2021). In Europe, the limit was set to 0.2% of Δ^9^-THC by the European Union in Regulation (EU) No. 1307/2013 in order to prevent the cultivation of illicit drug-type cannabis in hemp fields. However, in some countries, e.g., the Czech Republic, the limit increased up to 1%. On this basis, *Cannabis sativa* L. may be classified according to the variety or cultivar in relation to its Δ^9^-THC content, differentiating in hemp and cannabinoid-rich plants. Hemp is associated with low cannabinoid content and is used for industrial purposes. On the contrary, cannabinoid-rich plants are categorized depending on the amount of Δ^9^-THC in low THC content (<0.2%) and high THC content (>0.2%). Low-THC content cannabis is considered legal, and in many countries, its use for medical reasons is accepted, while high-THC content cannabis is deemed an illicit drug-type cultivation and consequently professed as a banned practice in several countries. Nevertheless, cannabis plants containing a higher content of THC may be cultivated, and even used for medical purposes, provided the obtention of specific permissions by competent authorities.

For this reason, in the last decade, many validated laboratories have emerged for forensic and legal purposes and quality control of the cannabis industry (Hädener et al., 2019). The European Commission established an official method to determine THC content using gas chromatography (Sgrò et al., 2021). According to the mentioned method, an adequate amount of dried plant sample, with a moisture content between 8 and 13%, is extracted with hexane and analyzed using the gas chromatography/flame ionization detector (GC-FID). The injector port is set to 300°C, while the column, impregnated with a 5% non-polar phenyl–methyl–siloxane phase, is fixed to 260°C. Results admit a tolerance of 0.03 g per 100 g, which is equivalent to the 0.3% allowed by different countries (European Commission, 2018). For financial purposes, cannabis farmers must prove the suitability of the crop by putting their plants to the test using the official method specified by accredited laboratories (European Monitoring Centre for Drugs and Drug Addiction, 2020). Nevertheless, problems arise at this stage since some discrepancies are observed when different chromatographic techniques are employed (Raharjo and Verpoorte, 2004).

Over the years, the influence of the GC inlet temperature in the degradation of many analytes has been evaluated. Kluchinsky et al. (2002) reported the identification of the thermal degradation products of 2-chlorobenzylidenemalononitrile (CS) generated at elevated temperatures in a tube furnace by using GC-MS. Different furnace temperatures were examined, spanning from 300 to 900°C, maintaining the GC inlet at a lower temperature. Despite the analysis of the blank, a spiked sample that was not thermally treated caused the emergence of a potential degradation product of CS. However, the effect of the equipment on the stability of the precursor compound was not assumed. A comparatively recent study reported the thermal degradation of food contaminants using pyrolysis-GC/MS (Tian et al., 2017). In this case, the melting point of these chemicals was considered to set the pyrolytic conditions, and 250°C was selected to maximize the thermal degradation. The effect of the equipment inlet temperature was not contemplated yet again, probably due to the brief residence time of the sample in the injector. Notwithstanding, the thermolability of the chemical compound may be critical at this juncture and, therefore, should not be underestimated (Kovacevik et al., 2016). Concerning cannabinoids, many authors have reported before the potential conversion of cannabidiol (CBD) into Δ9-THC as a consequence of external factors such as pH, oxidative reaction, light, or temperature (Golombek et al., 2020; Vacek et al., 2021; Yangsud et al., 2021).

For many years, different researchers have referred to the degradation of cannabinoids due to elevated temperatures (Czégény et al., 2021), and some of them even mentioned the presence of thermal degradation products using gas chromatography (Hazekamp et al., 2005). However, this technique is currently used for the analysis and control of critical vegetal material, regardless of the potential deviations that may be caused due to an uncontrolled transformation of cannabinoids. For this reason, in the present work, the assessment of the effect of GC inlet temperature on the stability of cannabinoids is carried out, suggesting different solutions for quality assurance and quality control purposes.

## 2 Materials and methods

### 2.1 Reagents

All the reagents were of analytical grade. Unless otherwise specified, they were purchased from Sigma-Aldrich (Madrid, Spain). CBD, cannabidiolic acid (CBDA), Δ^9^-THC, tetrahydrocannabinolic acid (THCA), cannabinol (CBN), cannabielsoin (CBE), cannabigerol (CBG), cannabigerolic acid (CBGA), delta-8 tetrahydrocannabinol (Δ^8^-THC), cannabichromene (CBC), and cannabicyclol (CBL) were the cannabinoid standards used to confirm the tentatively identified degradation products. All of them were prepared at a constant molar concentration to facilitate the comparison among experiments. Squalane was used as an internal standard (IS) during the experiments. Furthermore, calibration curves were obtained for each cannabinoid employing squalane as the IS in the range of temperatures studied for the purpose of quantifying the degradation rate in the different studies performed throughout the article. Derivatization of cannabinoids was carried out using N,O-Bis(trimethylsilyl)trifluoroacetamide (BSTFA) and trimethylchlorosilane (TMCS) for the silylation reaction. Additionally, the isotope-labeled standard of CBD and CBD-d_3_ was utilized for further experiments.

Acetonitrile and ammonium formate from Scharlab (Barcelona, Spain) and Milli-Q water from Merck/Millipore (Darmstadt, Germany) were used as components of the chromatographic mobile phase. Helium gas (99.999% purity) from Carburos Metálicos S.L. Spain was used as a carrier gas in the GC-MS analyses.

### 2.2 GC-MS equipment

GC-MS analyses were performed in an Agilent GC 7890B series (Agilent Technologies Inc., Santa Clara, CA, United States) equipped with a 7693 autosampler and a 5877B mass detector. System control and data acquisition were achieved using Agilent GC MassHunter Workstation software (Version 7.0). Unknown Analysis software was used to identify the peak chromatograms using the NIST spectra library. Chromatographic separation was achieved using a Rxi-35Sil MS capillary column (15 m length, 0.25 mm internal diameter, and 0.25 μm film thickness) (Restek, Bellefonte, PA, United States).

### 2.3 HPLC-DAD equipment

Liquid chromatographic analyses were performed using an Agilent 1260 series (Agilent Technologies Inc., Santa Clara, CA, United States) equipped with a binary high-pressure pump for mobile phase delivery, an autosampler, and a diode array detector (DAD). The HPLC separation was conducted on an InfinityLab Poroshell 120 EC-C18 analytical column (150 × 2.1 mm, 2.7 μm) using as mobile phase 50 mM aqueous ammonium formate buffer adjusted to pH 3.75 and acetonitrile, the proportion being 90:10 for solvent A and 10:90 for solvent B, respectively. The chromatographic separation was accomplished under gradient elution mode using 85% of solvent B held for 11 min and raised to 100% for 5 min. The column was thermostatically controlled at 35°C, and the flow rate was set to 0.2 ml/min. A volume of 3 μL was injected, and quantitation was achieved at 210 nm. Data analysis was carried out using Agilent OpenLab ChemStation software.

### 2.4 Thermal degradation using gas chromatography

The evaluation of the injector port of the gas chromatograph was evaluated at different temperatures. For this purpose, working standard solutions of CBD, CBDA, THC, THCA, CBG, and CBGA were prepared individually at a constant molar concentration (127 µM) in hexane, which corresponds to approximately 40 mg/L, and analyzed by GC-MS, modifying the temperature of the injector gradually, within an interval of 250–350°C. All the analyses were carried out by duplicate.

Additionally, the derivatization of cannabinoids was accomplished in further experiments by silylation of the hydroxyl groups, replacing the active hydrogens with trimethylsilyl groups, to study the effect of this protection in the degradation reaction. This process is a routine procedure to analyze acidic cannabinoids by GC-MS, which go through a decarboxylation reaction due to the elevated temperature. For this reason, a constant molar concentration (127 µM) of the purified standard was derivatized as follows: the cannabinoid solution was evaporated to dryness and subsequently reconstituted with 150 µL of pyridine and 1350 µL of BSTFA:TMCS (98:2 v/v). In the case of CBD, the isotope labeled standard (CBD-d_3_) was added prior to evaporation at a concentration of 10 mg/L to correct the potential random errors associated to sample preparation. Afterward, the mixture was incubated during 1 h at 37°C to complete the derivatization reaction, analyzing the silylated cannabinoids by GC-MS using the aforementioned method.

A supplementary trial was performed to irrevocably prove how the injector port produces the degradation of cannabinoids. To this end, the stability of the isotope-labeled standard of CBD (CBD-d3) was evaluated using squalane as the IS. Therefore, CBD-d_3_ and squalane were prepared in hexane at a constant concentration of 127 μM and 20 mg/L, respectively.

### 2.5 External influence of temperature

Once the effect of the injector temperature was evaluated, the effect of time on the potential degradation of CBD was studied using an external oven device. Therefore, the oven was set to 220°C according to the characteristic parameters of CBD. This cannabinoid presents the boiling point at 180°C and the flash point at 206°C (Chemspider database, 2015; Olejar et al., 2021). In this sense, the selected temperature is adequate for the assessment of CBD degradation. The experiment was carried out by duplicate, and the treated CBD was analyzed using HPLC-DAD, avoiding the effect of temperature since liquid chromatography was used, thus observing only the effect of the external oven temperature. For this reason, 25 mg of CBD were encapsulated in sealed vials and heated at 220°C for different times, ranging from 0.5 to 60 min. Additionally, a control sample, which corresponds to a sample without heating treatment, was analyzed to set the initial conditions. Finally, the resulting heated samples were dissolved with the appropriate amount of methanol. In this case, CBD was prepared at a higher concentration (458 µM) to ensure the correct monitoring of the degradation products regarding the lower sensitivity of the detector.

Thereafter, samples obtained following this procedure were also analyzed using GC-MS. Although the effect of temperature is not avoided in this case, degradation products may be elucidated by mass spectrometry. On the other side, by comparison with the previous trial performed with GC-MS, the effect of the external oven could be elucidated by the difference in the results. Likewise, 25 mg of CBD was encapsulated in sealed vials to reach the same molar concentration used in the rest of the experiments (127 µM).

### 2.6 Kinetic study of the degradation/emergence of cannabinoids

The evaluation of the temperature effect over time allowed monitoring of the kinetics of the degradation/emergence of cannabinoids. To this end, 25 mg of CBD was encapsulated in sealed vials and heated for different time periods, ranging from 0.5 to 60 min. Moreover, a CBD sample without thermal treatment was analyzed to be considered as a blank. Finally, an appropriate amount of hexane was added to the vials to reach the same molar concentration used in the rest of the experiments (127 µM). The concentration results calculated as µM were plotted against time (X-axis) expressed in seconds in order to simplify the results. Thus, concentration (Y-axis) was modified according to the potential reaction order, as shown in Table 1. The reaction order was identified based on the best-fitted degradation kinetics, which correspond to the highest *R* ^2^ value.

**TABLE 1:** Kinetics rate equations.

	Zero-order	First-order	Second-order
Rate law	$-\frac{d\left[A\right]}{dt}=k$	$-\frac{d\left[A\right]}{dt}=k\left[A\right]$	$-\frac{d\left[A\right]}{dt}=k{\left[A\right]}^{2}$
Integrated rate law	$\left[A\right]={\left[A\right]}_{0}-kt$	$\left[A\right]={\left[A\right]}_{0}e^{-kt}$	$\frac{1}{\left[A\right]}=\frac{1}{{\left[A\right]}_{0}}+kt$
Units of rate constant (k)	µM/s	1/s	1/µM·s
Linear plot to determine k	[A] vs t	ln ([A]) vs t	$\frac{1}{\left[A\right]}\;vs.\;t$

Additionally, pseudo-order reactions were evaluated for some specific cannabinoids. These types of equations are common in analytes involved both in the emergence and degradation processes. This occurs when there is a high amount of the compound under consideration or when it is an intermediate product. Therefore, in this particular case, the concentration of these cannabinoids remains constant for a certain period of time (El Seoud et al., 2016).

## 3 Results and discussion

### 3.1 Assessment of the injector temperature effect on cannabinoid degradation

The official method sets an inlet temperature of 300°C to ensure the complete evaporation of the solvent, thus preventing sample loss during the injection. However, considering the effect of temperature on the degradation of cannabinoids, it may be assumed that the GC-inlet port may affect the determination of these analytes. Therefore, different cannabinoid standards were selected to evaluate the potential effect of the isoprenoid unit precursor in the degradation process and prepared at a constant molar concentration to easily compare between experiments. Thus, degradation or emergence of compounds may be performed simply with moles instead of mass by means of molecular weight. Working standard solutions of CBD, CBDA, THC, THCA, CBG, and CBGA were prepared individually at a constant molar concentration (127 µM) in hexane and analyzed using GC-MS, modifying the temperature of the injector gradually in 10°C intervals, spanning from 250 to 350°C. The resulting chromatograms of each cannabinoid at different temperatures were analyzed to identify the degradation products after the thermal conversion and to quantify the remaining cannabinoid concentration. Identification was confirmed using standards of cannabinoids.

**FIGURE 1:**
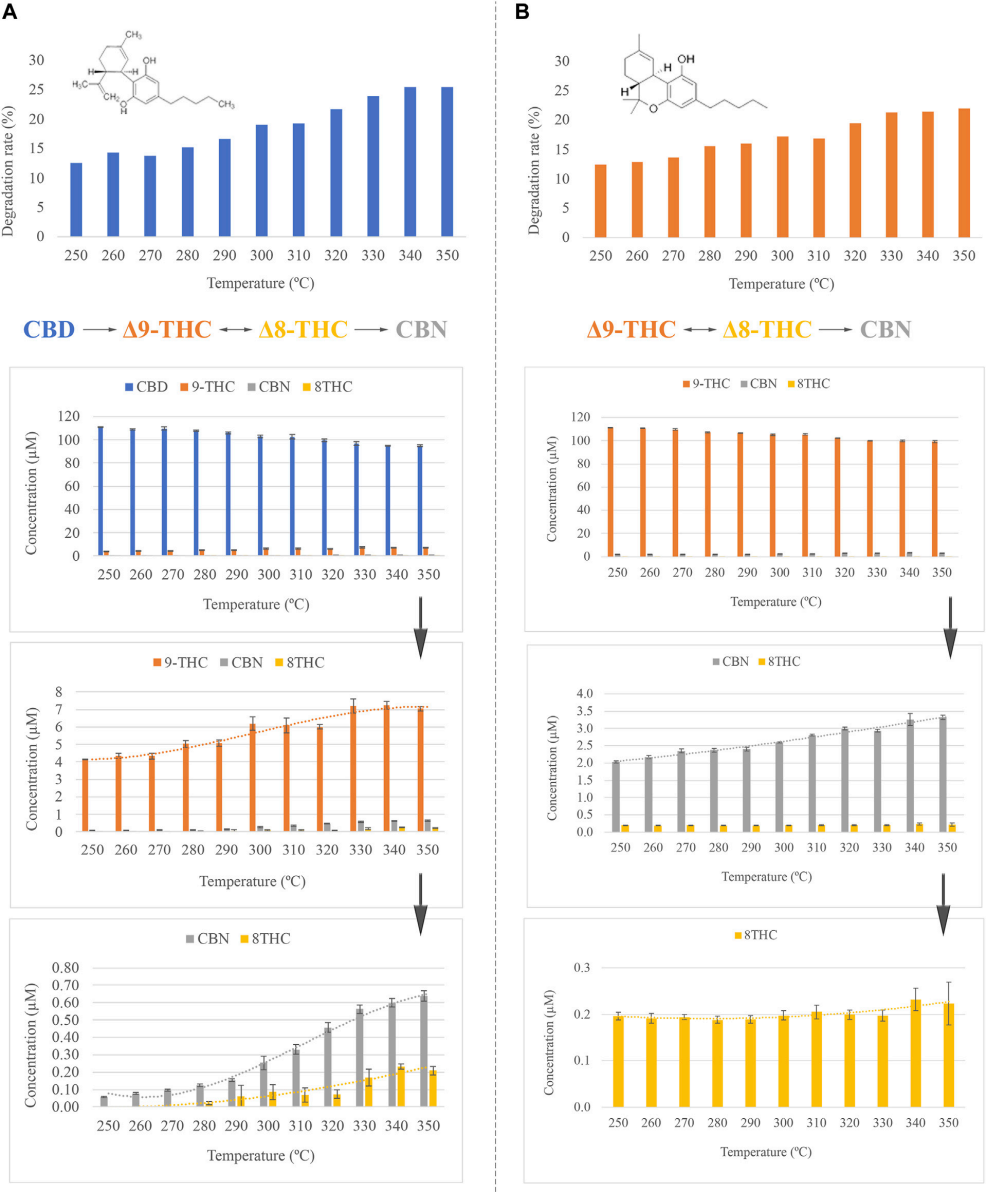
**(A)** Effect of the inlet temperature in CBD, degradation rate and concentration of CBD, Δ9-THC, CBN, and Δ8-THC expressed as µM. **(B)** Effect of the inlet temperature in Δ9-THC, degradation rate and concentration of Δ9-THC, CBN, and Δ8-THC expressed as µM.

A slight decline was observed in the concentration of CBD while the temperature of the GC injector increased. Thereby, the gradual degradation of CBD with temperature produces a reduction of up to 20% of the initial concentration when the temperature of the injector is set to 300°C (Figure 1A), which is the temperature used in the official method. However, as can be observed in Figure 1A, partial degradation of CBD is also observed at lower temperatures. At the same time, additional compounds gradually appear in the chromatogram, increasing their concentration with temperature. According to the mass spectra and the retention time of the compounds, the conversion of CBD into Δ^9^-THC, CBN, and Δ8-THC is produced during the volatilization of the sample in the injector port. Furthermore, Figure 1A shows the effect of the temperature on the emergence of these thermal degradation products. Thereby, Δ^9^-THC is largely produced in contrast to the rest of the cannabinoids. However, it is worth mentioning that although this is the main degradation product of CBD, the concentration slightly increases from 4 µM to 7 μM, resulting in an emergence percentage of 70%. On the contrary, CBN concentration increases from 0.06 µM to 0.64 µM, which corresponds to an appearance boosting of 1000%. This may be explained by the fact that CBN is a degradation product of Δ^9^-THC, thus reducing the appearance for this last analyte. The increase in the amount of CBN was quantified employing a calibration curve previously obtained for this compound in the presence of squalane as the internal standard. Additionally, the emergence of Δ^8^-THC, which has been previously associated with the degradation of Δ^9^-THC, was also observed. Some authors have previously pointed to the effect of light as the source of this conversion (Yangsud et al., 2021). Nevertheless, it is not unlikely that this transformation could take place at the GC inlet, considering the conditions of pressure and temperature.

In the case of Δ^9^-THC, as was expected according to the CBD results, the effect of the temperature of the GC inlet generates the degradation of Δ^9^-THC into CBN and Δ^8^-THC (Figure 1B). Naturally, the concentration of Δ^9^-THC decreases while the concentration of CBN increases. At the conditions reported in the official method, the concentration of Δ^9^-THC is reduced by 17.2% of the initial concentration. This loss was quantified employing a calibration curve previously obtained for Δ^9^-THC in the presence of squalane as the internal standard. Interestingly, the concentration of Δ^8^-THC remains constant over the complete temperature range. This behavior may be associated with two different explanations: contamination of the Δ^9^-THC standard by this cannabinoid or, perhaps, Δ^8^-THC might be subsequently degraded to some other compound. Considering the observed low concentration of this cannabinoid, any degradation product could not be detected, and, therefore, this is dismissed.

Additionally, the effect of the GC inlet temperature on the potential degradation of CBG was evaluated. As a result, it was observed a slight conversion of CBG into CBC (Supplementary Figure S1). In comparison to the other standards, the initial concentration of CBG is reduced by 35.6% at 250°C, which indicates the greater thermolability of this cannabinoid. Nevertheless, this degradation remains almost constant over the complete range of temperatures, dropping about 50% at 300°C.

**FIGURE 2:**
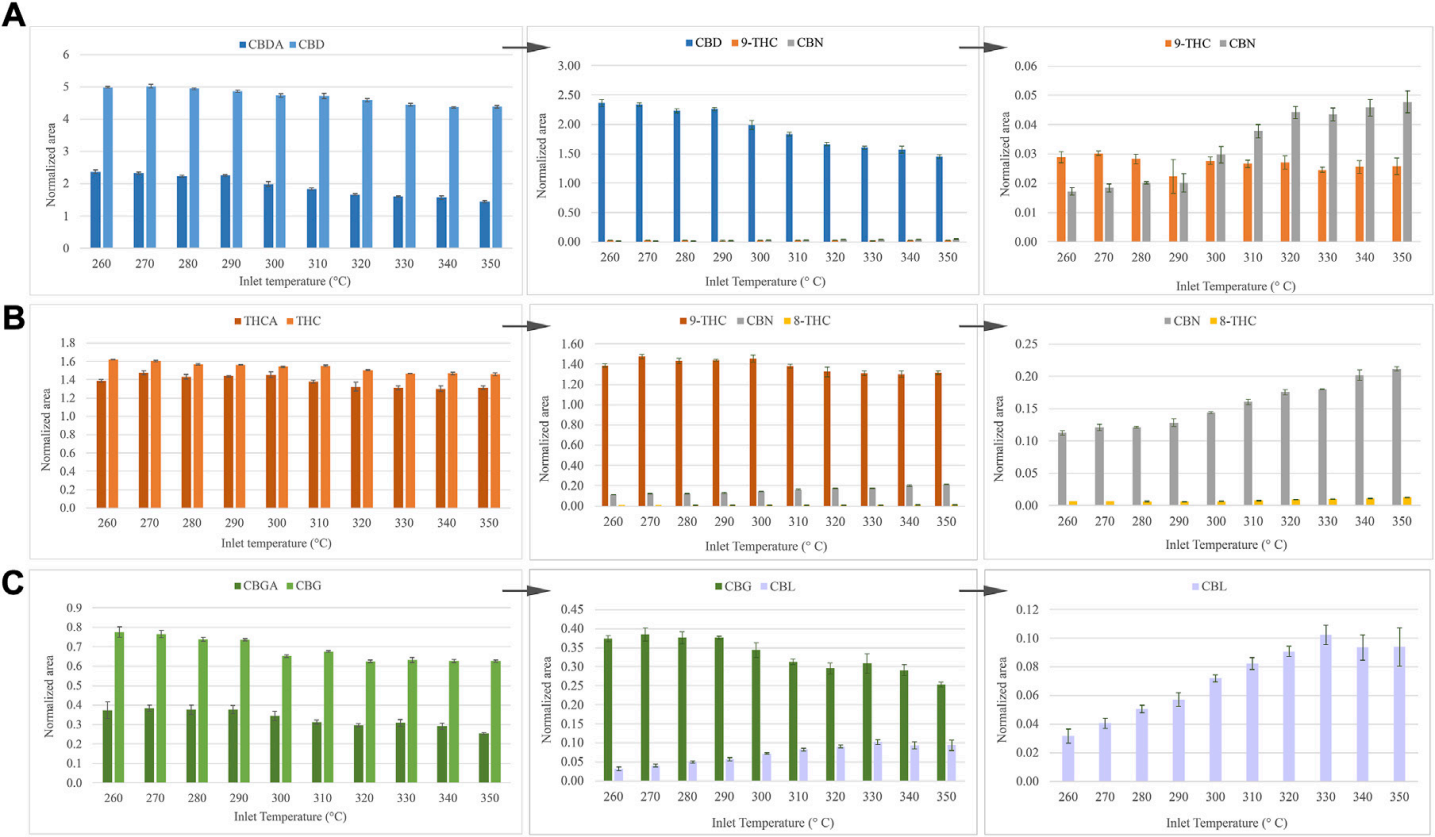
Effect of temperature inlet in the decarboxylation of acidic cannabinoids. **(A)** CBDA, **(B)** THCA and **(C)** CBGA.

One of the main drawbacks associated with the determination of cannabinoids with GC-MS is precisely related to the temperature of the injector port. The decarboxylation of the carboxyl group of acidic cannabinoids is produced by the effect of the temperature, limiting the direct analysis of these analytes by gas chromatography. Notwithstanding, the acidic form of each standard was also directly injected to evaluate the behavior of these compounds during the injection in GC-MS. Since CBDA and CBD were injected with the same molar concentration, assuming a total decarboxylation, the analytical signal in both cases should be equivalent. However, as is depicted in Figure 2A, an incomplete decarboxylation of the acidic cannabinoid in its neutral form is observed. Likewise, Δ^9^-THC and CBN appear as thermal degradation products of CBDA, as was observed for CBD. However, Δ^8^-THC was not detected in this case, possibly due to the addition of the initial decarboxylation step from CBDA to CBD, which reduces the availability of the latter compound. Regarding THCA, nearly all the acidic cannabinoid is transformed into its neutral form, in addition to CBN and Δ^8^-THC (Figure 2B). In contrast to what occurred with CBDA, a slight increase of Δ^8^-THC is observed with temperature. This result may be plausible considering the hypothesis of the additional decarboxylation step from the acidic cannabinoid. In the case of CBGA, an incomplete decarboxylation into CBG is observed, giving CBL as a degradation product (Figure 2C). Some researchers have previously reported the thermal cyclization of CBC to CBL, which may explain the observed results, bearing in mind the degradation of CBG into CBC (Caprioglio et al., 2019; Grafström et al., 2019).

Despite the problems encountered in the direct analysis of acidic cannabinoids, GC is a well-established technique since different derivatization processes have been proposed to overcome this issue (García-Valverde et al., 2020). However, this process is avoided in many instances since it involves some additional steps of sample treatment, and liquid chromatography is preferred. Notwithstanding, different chemicals have been used as modifiers of cannabinoids: N,O-bis (trimethylsilyl) trifluoroacetamide (BSTFA), trimethylchlorosilane (TMCS), N-methyl-N-(trimethylsilyl) trifluoroacetamide (MSTFA), or N-tert-butyldimethylsilyl-N-methyltrifluoroacetamide (MTBSTFA) (Cardenia et al., 2018). The first two derivatizers were the selected modifiers in the present work. The obtained results are depicted in Supplementary Figure S2, where it is demonstrated that the protection of the hydroxyl groups, both for neutral and acidic cannabinoids, prevents the degradation of cannabinoids caused by elevated temperatures at the injector port.

In this context, it seems contradictory that the official method, established by the Commission Regulation of the European Union, conducts the quantification of Δ^9^-THC content in hemp varieties using GC-FID without previous derivatization. In this regard, it is important to emphasize that the derivatization step in the analysis of cannabinoids should be a mandatory process and avoiding this procedure may lead to inaccurate results.

Finally, the evaluation of the effect of temperature in CBD-d_3_ confirmed what was previously observed. The injection of this standard produces the emergence of two additional peaks with m/z 302.2 and 298.2 (Supplementary Figure S3C), which correspond to the respective isotope labeled degradation products, namely, Δ^9^-THC-d_3_ and CBN-d_3_. This fact corroborates the degradation of this cannabinoid in the GC system since contamination from these two new compounds is discarded. Supplementary Figure S3A shows the performance of CBD-d_3_ over the range of temperatures, plotting the normalized area by correction with the IS area.

### 3.2 Effect of time on the degradation of cannabinoids

**FIGURE 3:**
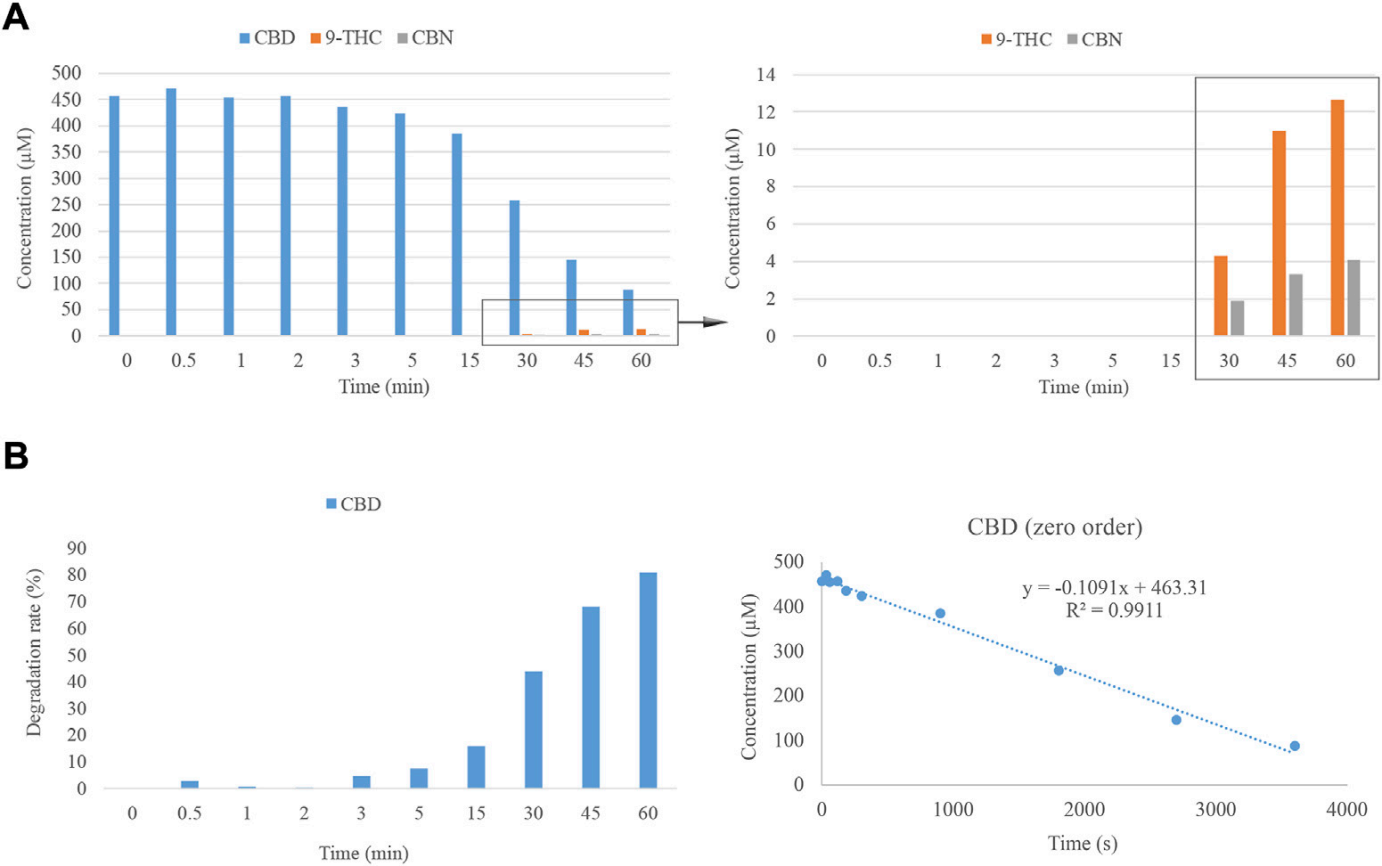
**(A)** Degradation of CBD considering the effect of time. **(B)** Degradation rate of CBD across the time interval under consideration at 220°C and zero-order plot for the degradation of CBD as a function of heating time.

Despite the brief residence time of the standards in the GC injector, some other processes require a longer period of time at an elevated temperature, such as the ones occurring in the pharmaceutical or food industry (Farjana et al., 2018; Priyadarshini et al., 2019; Tan et al., 2020). For this reason, the evaluation of the effect of time on the degradation of CBD was accomplished using HPLC-DAD. To this end, an external oven was employed to monitor the evolution of the degradation process over time, maintaining the temperature at 220°C for different times, spanning from 0.5 to 60 min. As was observed in the previous experiments, the degradation of CBD due to the effects of temperature results in the emergence of Δ^9^-THC and CBN. However, longer times than 30 min are required to observe additional breakdown products of CBD (Figure 3A). In this case, in contrast to the results observed at the GC inlet, Δ^8^-THC was not identified as a degradation product. However, this fact could be justified since the sensitivity of the detector may hinder the detection of this cannabinoid which, as was observed in the previous trial, is produced at a very low concentration. Additionally, considering the degradation rate as a way of comparison with the initial concentration of CBD, the amount of this cannabinoid remains nearly constant after 2 min of thermal treatment. However, after 60 min, the degradation of CBD is over 80% (Figure 3B). This last point must be taken under consideration when cannabinoids are thermally processed in the pharmaceutical or food industry.

In such a way, the better performance of liquid chromatography for the analysis of cannabinoids is demonstrated since side effects of temperature are avoided, and intricate derivatization processes are not required. However, mass spectrometry provides certain benefits, such as increased sensitivity, which allow monitoring of the degradation products at low concentrations.

On the other hand, the kinetics of the degradation reaction was evaluated, plotting the concentration of CBD against time to determine the zero-order reaction. The equation and *R* ^2^ for this order of the reaction are reported in Figure 3B. The constant rate (k) of this equation is equal to -0.1091 μM s^−1^, which corresponds to a degradation rate of 34.31 μg/L · s^−1^.

### 3.3 Evaluation of cannabinoid degradation by temperature accumulative effects

**FIGURE 4:**
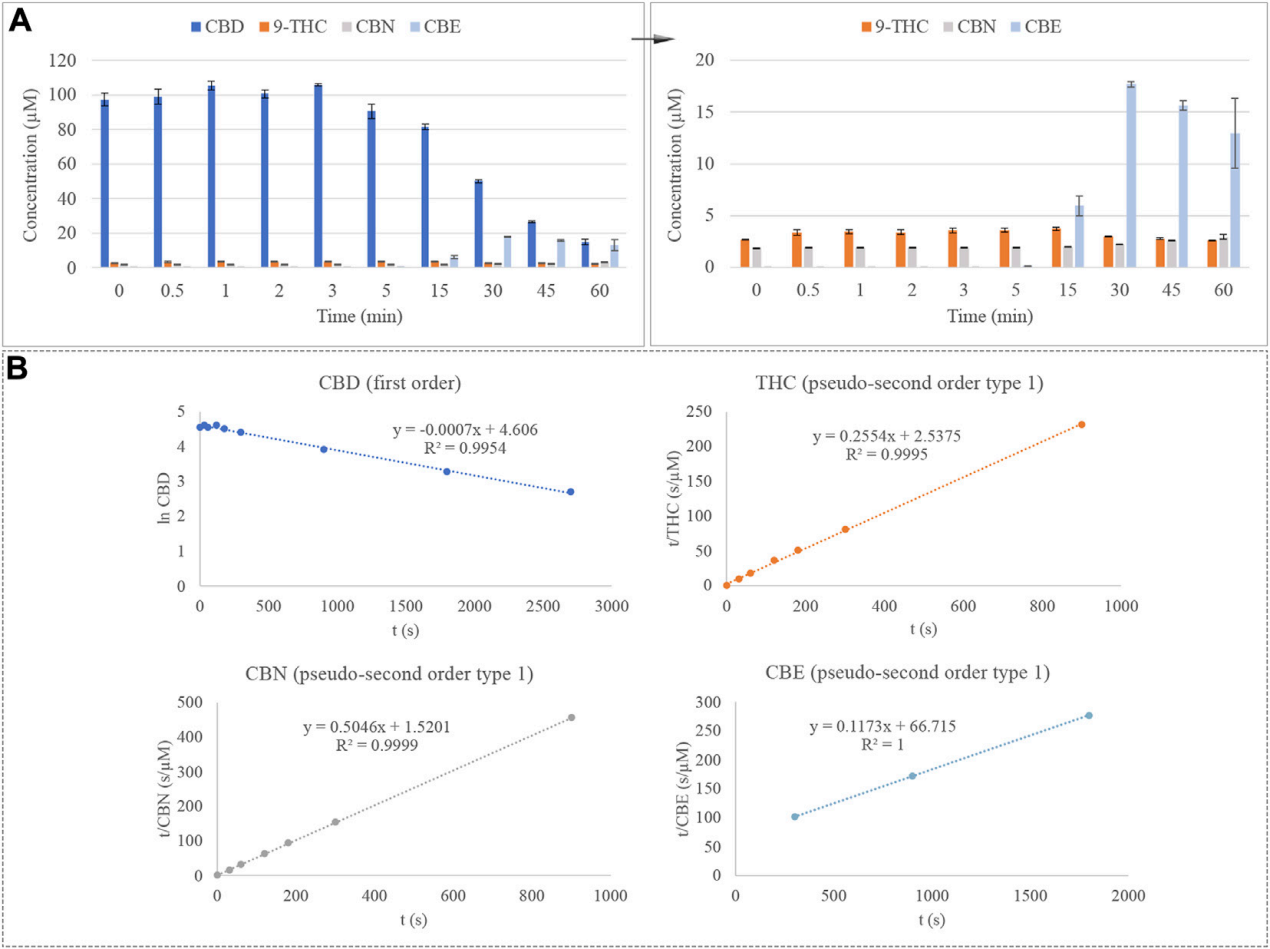
**(A)** Degradation of CBD considering the combined effect of time and temperature and emergence of Δ9-THC, CBN, and CBE. **(B)** Kinetic plots for the degradation of CBD under combined conditions and emergence of Δ9-THC, CBN, and CBE.

Once the effect of temperature and time were evaluated, a step forward was performed by combining both effects. For this purpose, the CBD standard was introduced into an external oven for a wide range of time at a setting temperature and, thereafter, analyzed using GC-MS, comprising the effect of the GC inlet temperature. The degradation of CBD under these conditions is depicted in Figure 4A. Once again, Δ^9^-THC and CBN emerge as degradation products of this cannabinoid. However, as was observed in the evaluation of the effect of time, Δ^8^-THC does not come up after analysis of thermally treated CBD, suggesting that the influence of the external oven may be altering the degradation reaction of this cannabinoid. This time, the emergence of CBE was noticed by mass spectrometry, which has been previously reported as a natural degradation product of CBD (Berman et al., 2018).

On the other hand, the kinetics of the degradation reaction of CBD was evaluated, plotting the natural logarithm (ln) of the concentration of CBD against time to determine the first-order reaction. In the case of Δ^9^-THC and CBN, the emergence reaction of these analytes adjusts better to equations of pseudo-second-order kinetic, which can be defined by plotting t/CBD concentration against time. The equation and *R* ^2^ for this order of reaction are reported in Figure 4B. The constant rate (k) of the CBD equation is -0.0007 s^−1^, which corresponds to a degradation rate of 0.0007 µM or 0.22 μg/L per second. Additionally, the emergence reaction of Δ^9^-THC, CBN, and CBE is depicted in Figure 4B. According to their constant rates, the velocity with which CBN is produced is 6 times higher than the emergence reaction of Δ^9^-THC, 0.1675 s^−1^ μM^−1^ against 0.0257 s-^1^ μM^-1^ for CBN and Δ^9^-THC, respectively. This fact makes sense since CBN is generated by the degradation of an intermediate compound, which is, therefore, presented in excess and is favoring the production of CBN. On the contrary, CBE emerges at a much slower rate than the Δ^9^-THC and CBN, being produced at 0.0002 s^−1^ μM^−1^.

## 4 Conclusion

Cannabinoids are influenced by different external factors such as pH, oxidation, light, and temperature. The latter is evidenced with acidic cannabinoids, which experience a decarboxylation process due to thermal treatments. Additionally, some other researchers have previously mentioned the conversion of Δ^9^-THC into CBN due to the aging of the plant (Dujourdy and Besacier, 2017; Meija et al., 2021). In this context, our study explores the effect of temperature in the GC injector port. Despite the brief residence of cannabinoids in the GC inlet during sample injection, the elevated temperature makes this time enough to degrade these compounds. Thus, the experiments carried out in the present work demonstrate the degradation of CBD into Δ^9^-THC and its subsequent conversion to CBN. Additionally, the emergence of CBC as a degradation product of CBG was observed. This fact hinders the determination of cannabinoids by GC, leading to altered results. However, this is the instrumental technique described in the official method established by the European Commission for the determination of the THC content; hence, a controversy arises since this method is employed by many laboratories to classify cannabis samples into legal or, by contrast, illicit drug–type cannabis. There is a great deal at stake since many agricultural subsidies depend on these analytical results, which might economically affect a company devoted to cannabis or hemp cultivation. For this reason, the evaluation of the stability of derivatized cannabinoids was also performed, demonstrating the absence of degradation products after their injection in the equipment, regardless of the injector temperature. On this basis, it is recommended to include a derivatization step prior to the injection for the protection of labile groups to assure the proper determination of cannabinoids by GC.

Additionally, the exposure of cannabinoids to elevated temperatures for a longer period was evaluated. This effect may be comparable to thermal treatments produced in the pharmaceutical or food industry, which are subjected to lengthy processes. An external oven was employed to this end, observing the appearance of new degradation products of CBD, as is the case of CBE. The evaluation of temperature and time simultaneously allowed the kinetic study of the degradation reactions. According to the constant rate of CBN and Δ^9^-THC, it may be concluded that the higher production per time of CBN is ascribed to the fact that Δ^9^-THC is an intermediate compound produced from the degradation of CBD.

## Data Availability

The original contribution presented in the study are included in the article/Supplementary Material; further inquiries can be directed to the corresponding author.
