# Supplementary material for: Effect of temperature in the degradation of cannabinoids: From a brief residence in the gas chromatography inlet port to a longer period in thermal treatments
Source: Front Chem. 2022 Nov 1;10:1038729. doi: 10.3389/fchem.2022.1038729 (PMC9664148; doi:10.3389/fchem.2022.1038729)
Supplement: Supplementary file 1 [file DataSheet1.docx]

**Effect of temperature in the degradation of cannabinoids: from brief residence in the GC inlet port to longer period in thermal treatments**

María Teresa García-Valverde^1^*, Carolina Sánchez-Carnerero Callado^1^, María del Carmen Díaz-Liñán^1^, Verónica Sánchez de Medina^1^, Jesús Hidalgo-García^1^, Xavier Nadal^1^, Lumı́r Hanuš^2^, Carlos Ferreiro-Vera^1^

^1^ Phytoplant Research S.L.U., The Science and Technology Park of Córdoba-Rabanales 21, Córdoba, Spain

^2^ Institute for Drug Research, School of Pharmacy, Faculty of Medicine, Hebrew University, Ein Kerem Campus, Jerusalem, Israel

Supporting Information

**Number of pages: 3**

**Figures S1, S2 and S3**

*** Correspondence:**

María Teresa García-Valverde. E-mail: mt.garcia@phytoplant.es


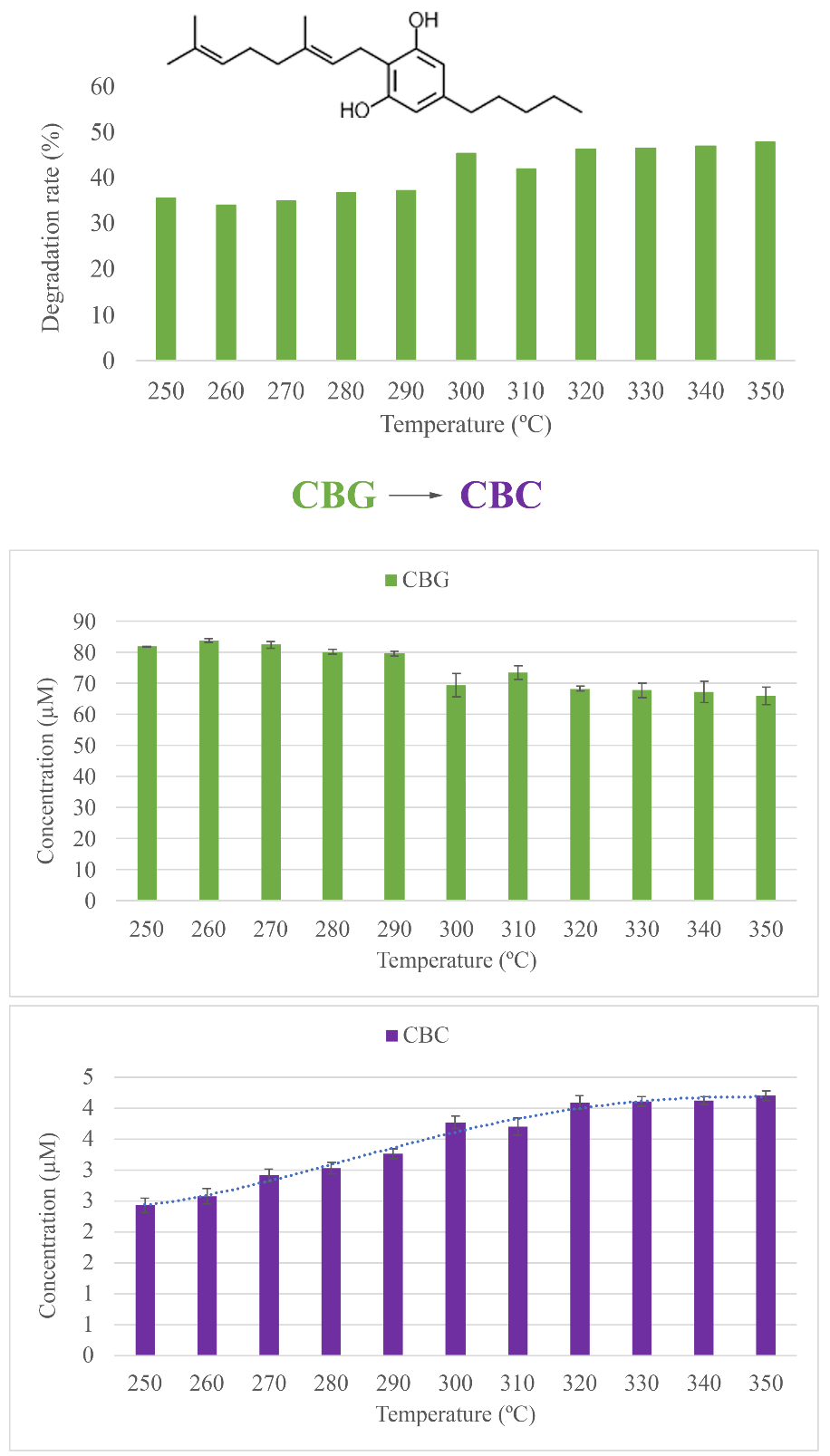


**Figure S1.** Effect of inlet temperature in CBG, degradation rate and concentration of CBG and CBC, expressed as µM.


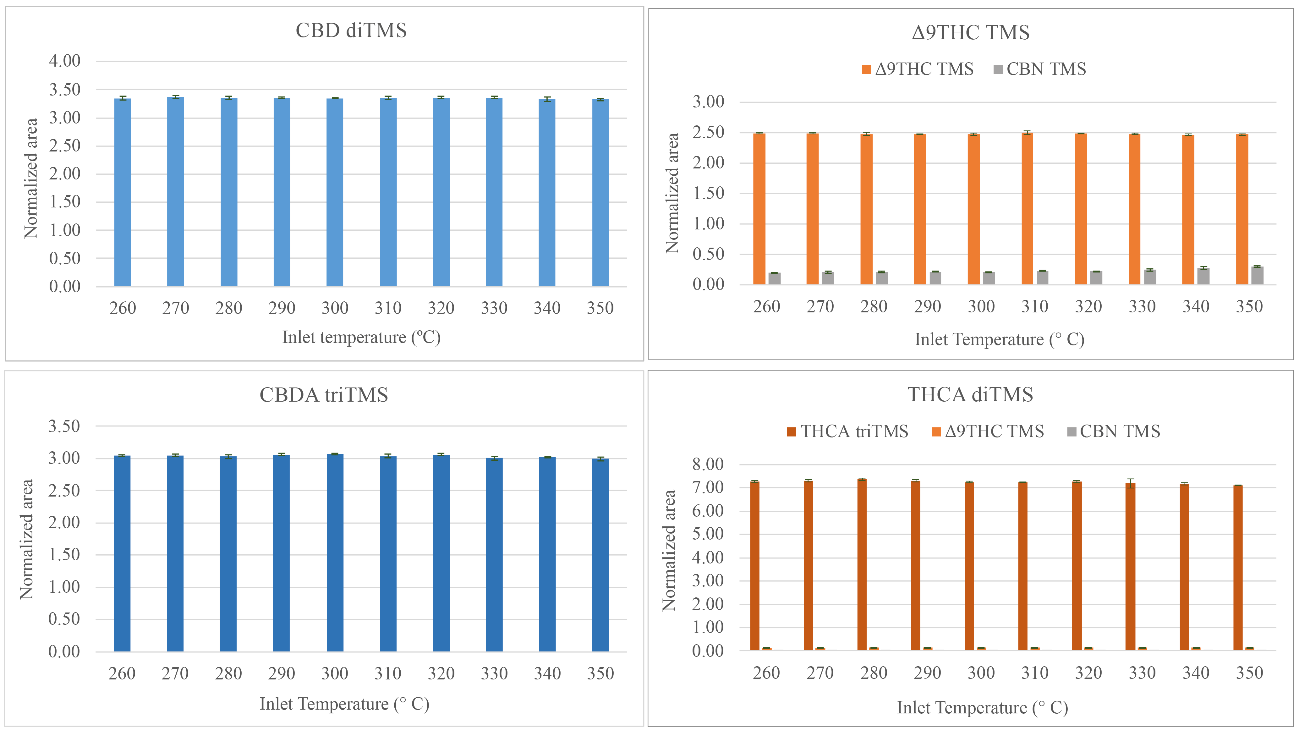


**Figure S2.** Effect of hydroxyl groups protection after silylation derivatization.


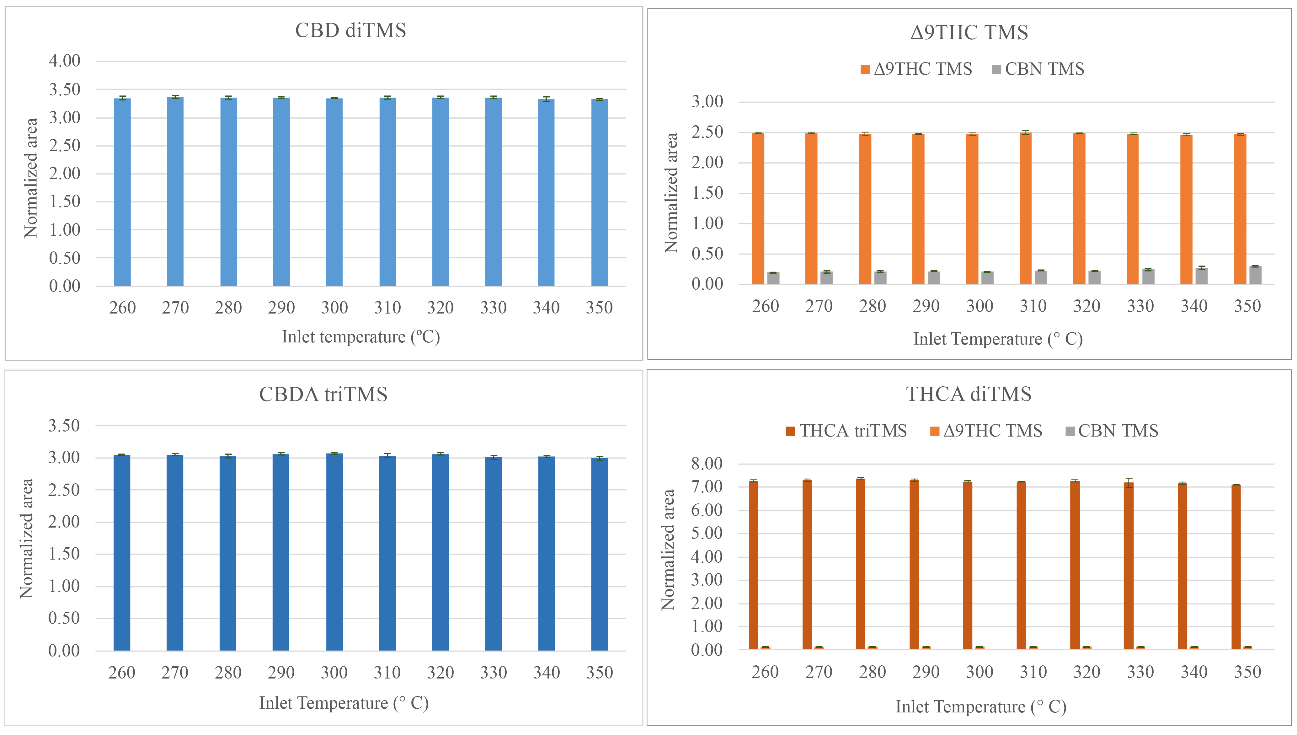


**Figure S3.** A) Normalized area of CBD-d3 and its degradation products Δ9-THC-d3 and CBN-d3 at different GC inlet temperatures. B) Analytical chromatograph of the CBD-d3 standard injected at 350 ºC. C) Mass spectra of CBD-d3, Δ9-THC-d3 and CBN-d3, subtracted from the analytical chromatograph.
